# Decreasing prevalence or increase in unregistered cases of bulimia nervosa in children and adolescents in Germany? A comparison using representative claims data pre- vs. intra-COVID-19 pandemic

**DOI:** 10.1007/s40519-025-01738-z

**Published:** 2025-03-21

**Authors:** Jule Leickert, Stephan Zillmer, Christian J. Bachmann, Annika Vivirito, Dirk Enders, Josephine Pintsch, Christoph U. Correll, Charlotte Jaite

**Affiliations:** 1 Department of Clinical Psychology and Psychotherapy in Childhood and Adolescence, University of Hildesheim, Universitätsplatz 1, 31141 Hildesheim, Germany; 2 Department of Child and Adolescent Psychiatry, Psychosomatic Medicine and Psychotherapy, Charité - Universitaetsmedizin Berlin, Corporate Member of Freie Universitaet Berlin, Humboldt Universitaet Zu Berlin, and Berlin Institute of Health, Berlin, Germany; 3 Department of Child and Adolescent Psychiatry, University of Ulm, Ulm, Germany; 4 Ingef - Institute for Applied Health Research Berlin Gmbh, Berlin, Germany; 5 Department of Psychiatry and Molecular Medicine, Zucker School of Medicine at Hofstra/Northwell, Hempstead, NY USA; 6 Center for Psychiatric Neuroscience, Feinstein Institute for Medical Research, Manhasset, NY USA; 7 German Center for Mental Health (DZPG), Partner Site Berlin, Berlin, Germany; 8 German Center for Child and Adolescent Health (DZKJ), Partner Site Berlin, Berlin, Germany

**Keywords:** Bulimia nervosa, COVID-19, Children, Adolescents, Germany, Epidemiology, Health care services

## Abstract

**Purpose:**

The aim of this study was to analyze data of children and adolescents in Germany insured according to legal requirements (statutorily insured) regarding epidemiology, comorbidities, and care of bulimia nervosa (BN) pre- vs. intra-COVID-19 pandemic.

**Methods:**

The study is based on anonymized claims data of 10–17.9 years old children and adolescents statutorily insured in Germany from the InGef Berlin GmbH research database. The database combines data of more than 50 statutory health insurances and is representative of the German population. Prevalence, (quarterly) incidence, comorbidities and in- and outpatient treatment of BN (ICD-10: F50.2/F50.3) pre-COVID (01/2018-03/2020; N = 282,711) vs. intra-COVID (04/2020-12/2021; N = 282,738) was compared using descriptives and χ^2^ tests, Welch-tests and interrupted time series analysis. The analysis was stratified by age groups (children: 10–13 years; adolescents: 14–17 years).

**Results:**

Prevalence of BN was 0.09% pre-COVID and 0.07% intra-COVID (OR = 0.78 [0.65, 0.93]). After pandemic onset, a positive trend in the quarterly incidence among adolescents was observed (*p* = .016). Outpatient visits to general practitioners decreased (OR = 0.59 [0.35, 0.98]).

**Conclusion:**

The observed decline in diagnosed and treated BN cases and the positive trend in quarterly incidence could be attributed to an increase in unregistered cases due to the overburdened care situation that emerged with the onset of the COVID-19 pandemic. Researchers and healthcare providers need to be aware of the potential for a backlash and deterioration/chronification of BN symptoms in children and adolescents.

*Level of evidence* No level of evidence.

**Supplementary Information:**

The online version contains supplementary material available at 10.1007/s40519-025-01738-z.

## Introduction

A series of infection prevention measures, including social distancing, curfews and school closures were implemented in March 2020 in response to the onset of COVID-19 in Germany [1]. Several studies have shown the negative effects of these restrictions on childrens’ and adolescents’ mental health and quality of life (e.g. [2]). The development of eating disorders (EDs) was of particular interest, as the restrictions caused loss of control and structure [3], which may increase the risk of developing, or worsening EDs. Consequently, an increase in incidence of EDs in female adolescents in Germany by 51% from 2019 to 2022 was reported [4]. Despite a 14% decline in incidence from 2021 to 2022 among 10–17-year-olds, ED incidence remained higher than pre-COVID. A systematic review [3] of studies published between November 2019 to October 2021 reported on COVID-related deteriorations in ED symptoms (e.g. exercise, binge eating) in people with anorexia nervosa (AN), bulimia nervosa (BN), binge-eating disorder (BED), and other specified feeding and ED, as well as a diagnostic specific decrease in BMI for patients with BN during lockdown [5]. Furthermore, Devoe et al. found an increase of 83% in pediatric hospitalizations due to EDs when averaging international admission rates in children and adolescents (probably from Australia, Canada, United Stated, Spain, New Zealand, among others) [3]. The increase in adult admission rates was lower, averaging only 16%. However, it was not clearly reported which studies were included in the average numbers. A meta-analysis [6] also found significant increases in self-reported symptom prevalence by 39–80% and 42–74% in AN and BN, respectively, but also decreases in symptom prevalence during COVID-19. Although, the odds of a symptom prevalence increase were higher compared to a decrease within subjects. Significant deteriorations in ED symptoms were specifically found in AN. Nevertheless, most studies did not differentiate their data (analyses) by specific ED diagnoses or solely focused on AN [3, 6], given the potentially fatal consequences of AN. Herpertz-Dahlmann et al. [7] found an increase of hospitalizations due to (atypical) AN by 40% in female youth in Germany. In contrast, international results on BN have indicated a decline in the proportion of BN among ED-related hospitalizations in Canada. Pre-COVID, 10.3% of youth aged 10–19 years hospitalized for an ED were diagnosed with BN, while the proportion decreased to 8.3% between March and August 2020, decreasing to 6.4% between September 2020 and March 2021 [8]. German inpatient departments of child and adolescent psychiatry also recorded a 18.1% decrease of BN hospitalizations, but in pediatric inpatient departments rates increased by 32.1% [9]. While research on EDs in general and AN in particular during COVID-19 has yielded comparable results regarding increased prevalences and incidences as well as deteriorations, studies on BN are scarce and reveal divergent findings. Therefore, the aim of this study was to contribute to existing research about specific EDs during COVID-19 by also including data of the epidemiology, comorbidity and care of children and adolescents with BN in Germany drawn from a representative database.

## Method

Anonymized claims data (secondary data) were studied using the InGef Berlin GmbH research database comprising 4 million statutorily insured individuals in Germany annually [10]. This database is representative of the German population. In Germany, it is a legal requirement to have health insurance. There are two types of health insurance (statutory and private) with different providers to choose from [11, 12]. Approximately 90% of the population is covered by statutory health insurance. Children and adolescents are typically insured through their parents or legal guardians. Data from children and adolescents aged 10.0–17.9 years insured pre-COVID (01/2018-03/2020; N = 282,711) and intra-COVID (04/2020-12/2021; N = 282,738) with a maximum insurance gap of 30 days were obtained (i.e., base population). Within the base population, children and adolescents diagnosed with BN (F50.2) or atypical BN (F50.3) coded according to ICD-10 [13] constituted the study population (= BN prevalence). Cases are only counted if the first ED diagnosis was BN among the following categories: AN, restrictive type (F50.00); AN, binge-eating/purging type (F50.01); other and unspecified AN (F50.08); atypical AN (F50.1); BN (F50.2); atypical BN (F50.3); other EDs (F50.8); ED, unspecified (F50.9), during the defined study period. For example, if the first diagnosis in the defined study period was other EDs and the patient subsequently received a diagnosis of BN, the patient would not be included in the study population. Prevalent children and adolescents with insurance and no ED diagnoses in the four quarters preceding each study period were defined as incident (= BN incidence). Incidence by quarter was also provided. The case definitions for prevalence and incidence included inpatient main and secondary diagnoses as well as outpatient confirmed diagnoses. We compared pre-COVID vs. intra-COVID data using descriptive analyses, χ^2^-tests, Welch-tests using Excel for Mac version 16.79.1. Interrupted time series analyses applying AutoRegressive Integrated Moving Average (ARIMA) model determined trends in quarterly incidence using SPSS for Mac version 29.0.0.0. Odds ratios (OR) for χ^2^-tests and Cohen’s d for Welch-tests (small effect: ≥ 0.2, medium effect: ≥ 0.5, large effect: ≥ 0.8) [14] were calculated to estimate effect sizes. We considered the following age groups: 10–13 years (referred to as “children”, pre-COVID n = 140,454 vs. intra-COVID n = 143,048) and 14–17 years (referred to as “adolescents”, pre-COVID n = 142,257 vs. intra-COVID n = 139,690).

Frequency of at least one psychiatric comorbidity (ICD-10: F00-F99) in children and adolescents with BN were compared pre- vs. intra-COVID.

Furthermore, the frequency of at least one outpatient visit (general practitioner, pediatrician, psychiatrist/neurologist, child and adolescent psychiatrist, gynecologist, gastroenterologist, endocrinologist/diabetologist, psychological psychotherapist, child and adolescent psychotherapist), the proportion with outpatient psychotherapy and prescription of antidepressants (ATC: N06A) or antipsychotics (ATC: N05A), with at least one hospitalization (child and adolescent psychiatry, general psychiatry, psychosomatics/psychotherapy, pediatrics), mean inpatient treatment duration, and mean duration until first outpatient contact after discharge (general practitioner, pediatrician, psychiatrist/neurologist, child and adolescent psychiatrist, psychological psychotherapist, child and adolescent psychotherapist) of children and adolescents with BN were compared pre- vs. intra-COVID. The analyzed health care services were billed based on the standardized evaluation scale (Einheitlicher Bewertungsmaßstab [EBM]) or pharmacy billing, which is mandatory for practitioners. The EBM defines the content of billable services provided by accredited practitioners and expresses the value of the defined service. Accredited practitioners can bill health insurance companies for these services, which means they are documented and consequently included in the InGef Berlin GmbH database. Due to data collection based on diagnostic codes, EBM or pharmacy billing (claims data), any treatment utilization in children and adolescents with BN reported cannot be attributed solely to BN. Alpha-level was set at 0.05 for all tests. Given the hypothesis-generating exploratory analyses, we did not correct for multiple testing. As some analyses suffered from small sample sizes, changes from pre- to intra-COVID are reported in the text if the magnitude of the change was >  ± 50.0% or if the change yielded a *p* < 0.05. This was done to capture potentially clinically relevant changes, regardless of statistical power (however, for full psychiatric comorbidity results, see Electronic Supplementary Material 1).

## Results

### Prevalence and incidence

**Fig. 1:**
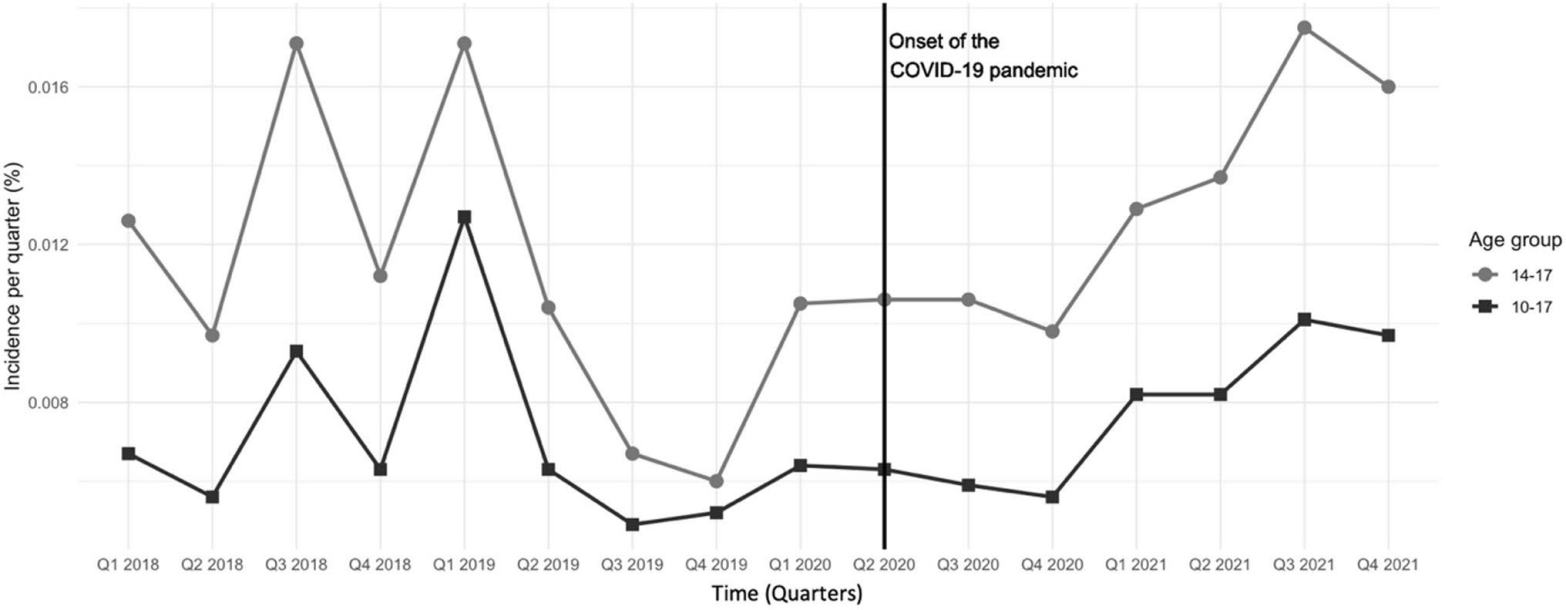
Quarterly incidence (%) of bulimia nervosa by age group before vs. during the COVID-19 pandemic

From pre-COVID to intra-COVID, the prevalence of BN within the base population declined significantly from 0.09% to 0.07% (− 22.0%; χ^2^(1) = 7.31, *p* = 0.0069) with reduced odds of a BN-diagnosis intra-COVID (OR = 0.78 [0.65;0.93]). The BN prevalence within the base population of adolescents declined significantly by 21.7% (χ^2^(1) = 5.98, *p* = 0.0145, OR = 0.78 [0.64;0.95]). Please refer to Table 1 for further details. Before the pandemic, the incidence of BN within the base population declined numerically by 0.001% per quarter (*p* = 0.0974). However, we found a significant positive change in slope by 0.002% among adolescents from pre- to intra-COVID (*p* = 0.0159) yielding a quarterly rise in the incidence by 0.001% from 04/2020 to 12/2021 within the base population. This increase in the quarterly incidence can be observed particularly from 10/2020 (Q4/2020) to 09/2021 (Q3/2021) *(*Fig. 1*)*. Quarterly incidence within the base population for children could not be analyzed due to low case numbers.

**Table 1:** Prevalence, incidence and comorbidities of bulimia nervosa in girls and boys pre- vs. intra-COVID, stratified by age groups

Age group	10–13 years				14–17 years				10–17 years			
	Pre- COVID	Intra-COVID	Δ%	OR [95%CI]	Pre-COVID	Intra-COVID	Δ%	OR [95%CI]	Pre-COVID	Intra-COVID	Δ%	OR [95%CI]
Prevalence, n (%)	38 (0.03)	32 (0.02)	− 17.34	0.83 [0.52;1.32]	230 (0.16)	177 (0.13)	− 21.65	0.78* [0.64;0.95]	268 (0.09)	209 (0.07)	−22.01	0.78** [0.65;0.93]
Incidence, n (%)	34 (0.03)	25 (0.02)	− 27.78	0.72 [0.07;7.60]	136 (0.10)	120 (0.09)	− 10.11	0.90 [0.70;1.15]	170 (0.06)	145 (0.05)	−14.73	0.85 [0.68;1.06]
Psychiatric comorbidities, n (%)	33 (86.84)	29 (90.63)	+ 4.36	1.46 [0.32;6.67]	186 (80.87)	148 (83.62)	+ 3.40	1.21 [0.72;2.02]	219 (81.72)	177 (84.69)	+ 3.64	1.24 [0.76;2.02]
Anxiety and emotional disorders (F40, F41, F93)	9 (23.68)	19 (59.38)	+ 150.69	4.71** **[1.68;13.17]**	59 (25.65)	64 (36.16)	+ 40.96	1.64* **[1.07;2.51]**	68 (25.37)	83 (39.71)	+ 56.52	1.94*** **[1.31;2.86]**
Obsessive–compulsive disorder (F42)	–	< 5	–	–	8 (3.48)	8 (4.52)	+ 29.94	1.31 [0.48;3.57]	8 (2.99)	11 (5.26)	+ 76.32	1.81 [0.71;4.57]
Somatoform disorder (F45)	10 (26.32)	7 (21.88)	− 16.88	0.78 [0.26;2.37]	55 (23.91)	22 (12.43)	− 48.02	0.45** **[0.26;0.77]**	65 (24.25)	29 (13.88)	− 42.79	0.50** **[0.31;0.81]**
Disturbance of activity and attention (F90.0)	< 5	6 (18.75)	–	–	11 (4.78)	14 (7.91)	+ 65.38	1.71 [0.76;3.86]	13 (4.85)	20 (9.57)	+ 97.28	2.08* [1.01;4.28]

Note. Bold text indicates a significant change

*CI* confidence interval, *n* number of cases, *F40* Phobic anxiety disorders; F41, Other anxiety disorders; F93, Emotional disorders with onset specific to childhood; Odds Ratios (OR) calculated as effect estimates based on the odds of the respective time period, change in the probability of occurrence by OR from pre- to intra-COVID; Δ%, %-change; * p < .05; ** p < .01; *** p < .001

### Psychiatric comorbidities

Intra-COVID, comorbid anxiety and emotional disorders (F40, F41, F93) increased significantly in the study population with BN by 56.2% (χ^2^(1) = 11.16, *p* = 0.0008, OR = 1.94 [1.31;2.86]) with higher increases in children than adolescents (+ 150.7%; χ^2^(1) = 9.22, *p* = 0.0024, OR = 4.71 [1.68;13.17] vs. + 41.0%; χ^2^(1) = 5.24, *p* = 0.0221, OR = 1.64 [1.07;2.51]). Comorbid attention-deficit/hyperactivity disorders (ADHD; F90.0) also increased significantly in the study population with BN (+ 97.3%; χ^2^(1) = 4.06, *p* = 0.0439; OR = 2.08 [1.01;4.28]), especially albeit only numerically in adolescents (+ 65.4%; χ^2^(1) = 1.70, *p* = 0.1927, OR = 1.71 [0.76;3.86]). Comorbid obsessive–compulsive disorders (OCD; F42) also showed notable increases in the study population with BN notably but only numerically (+ 76.3%; χ^2^(1) = 1.59, *p* = 0.2068, OR = 1.81 [0.71;4.57]). Somatoform disorders (F45) declined significantly in the study population (−42.8%; χ^2^(1) = 7.99, *p* = 0.0047, OR = 0.50 [0.31;0.81]), and especially in adolescents with BN (−48.0%; χ^2^(1) = 8.60, *p* = 0.0034; OR = 0.45 [0.26;0.77]). Finer-grained details can be found in Electronic Supplementary Material 1.

### Inpatient and outpatient care

The number of outpatient visits to general practitioners by children and adolescents with BN declined significantly intra-COVID (−7.5%; χ^2^(1) = 4.15, *p* = 0.0416; OR = 0.59 [0.35;0.98]), especially in adolescents (−7.9%; χ^2^(1) = 5.64, *p* = 0.0176, OR = 0.46 [0.24;0.88]). The number of outpatient visits to child and adolescent psychiatrists by children with BN increased substantially numerically intra-COVID (+ 72.7%; χ^2^(1) = 3.25, *p* = 0.0714, OR = 2.45 [0.92;6.58]). The prescription of antidepressants in children increased non-significantly (+ 66.3%; χ^2^(1) = 0.93, *p* = 0.3350, OR = 1.58 [0.52;6.51]). More details can be found in Table 2. The mean duration until the first outpatient visit to child and adolescent psychiatrists after discharge from inpatient treatment was 49.5 days longer intra-COVID vs. pre-COVID in children with BN, although this change was not statistically significant (+ 313.5%; t(5.20) = − 1.40, *p* = 0.2210, |d|= 1.06). Please refer to Table 3 for more details.

**Table 2:** Outpatient treatment in girls and boys with bulimia nervosa pre- vs. intra-COVID, stratified by age groups

Age group	10–13 years				14–17 years				10–17 years			
	Pre-COVID	Intra-COVID	Δ%	OR [95%CI]	Pre-COVID	Intra-COVID	Δ%	OR [95%CI]	Pre-COVID	Intra-COVID	Δ%	OR [95%CI]
Professional groups^1^, n (%)												
General practitioner	24 (63.16)	20 (62.50)	− 1.04	0.97 [0.37;2.57]	213 (92.61)	151 (85.31)	− 7.88	0.46* **[0.24;0.88]**	237 (88.43)	171 (81.82)	− 7.48	0.59* [0.35;0.98]
Pediatrician	22 (57.89)	19 (59.38)	+ 2.56	1.06 [0.41;2.76]	79 (34.35)	76 (42.94)	+ 25.01	1.44 [0.96;2.15]	101 (37.69)	95 (45.45)	+ 20.61	1.38 [0.95;1.99]
Child and adolescent psychiatrist	11 (28.95)	16 (50.00)	+ 72.73	2.45 [0.92;6.58]	86 (37.39)	75 (42.37)	+ 13.32	1.23 [0.83;1.84]	97 (36.19)	91 (43.54)	+ 20.30	1.36 [0.94;1.97]
Child and adolescent psychotherapist	25 (65.79)	15 (46.88)	− 28.75	0.46 [0.17;1.20]	112 (48.70)	84 (47.46)	− 2.54	0.95 [0.64;1.41]	137 (51.12)	99 (47.37)	− 7.34	0.86 [0.60;1.24]
Only outpatient psychotherapy, n (%)	22 (57.89)	18 (56.25)	− 2.84	0.82 [0.32;2.13]	121 (52.61)	–	+ 0.95	0.98 [0.66;1.45]	–	–	+ 1.32	0.96 [0.67;1.37]
At least one medication, n (%)	8 (21.05)	9 (28.13)	+ 33.59	1.47 [0.49;4.39]	80 (34.78)	62 (35.03)	+ 0.71	1.01 [0.67;1.53]	88 (32.84)	71 (33.97)	+ 3.46	1.05 [0.73;1.54]
Antidepressant, n (%)	5 (13.16)	7 (21.88)	+ 66.25	1.85 [0.52;6.51]	71 (30.87)	51 (28.81)	− 6.66	0.91 [0.59;1.39]	76 (28.36)	58 (27.75)	− 2.14	0.97 [0.65;1.45]
Antipsychotic, n (%)	< 5	< 5	–	–	22 (9.57)	19 (10.73)	+ 12.22	1.14 [0.60;2.17]	25 (9.33)	21 (10.05)	+ 7.71	1.09 [0.59;2.00]

Note. Bold text indicates a significant change

*CI* confidence interval, *n* number of cases; Odds Ratios (OR) calculated as effect estimates based on the odds of the respective time period, change in the probability of occurrence by OR from pre- to intra-COVID; Δ%, %-change; *p < .05; **p < .01; ***p < .001

^1^All BN patients had at least one outpatient contact pre- and intra-COVID

**Table 3:** Inpatient treatment in girls and boys with bulimia nervosa pre- vs. intra-COVID, stratified by age groups

Age group	10–13 years				14–17 years				10–17 years			
	Pre-COVID	Intra-COVID	Δ%	OR [95% CI] |d|	Pre-COVID	Intra-COVID	Δ%	OR [95% CI] |d|	Pre-COVID	Intra-COVID	Δ%	OR [95% CI] |d|
At least one hospitalization, n (%)	16 (42.11)	8 (25.00)	− 40.63	0.46 [0.16;1.28]	89 (38.70)	57 (32.20)	− 16.78	0.75 [0.50;1.14]	105 (39.18)	65 (31.10)	− 20.62	0.70 [0.48;1.10]
Professional groups, n (%)												
Pediatrician	7 (43.75)	< 5	–	–	34 (38.20)	16 (28.07)	− 26.52	0.63 [0.31;1.30]	41 (39.05)	20 (30.77)	− 21.20	0.69 [0.36;1.34]
Child and adolescent psychiatry	14 (87.50)	6 (75.00)	− 14.29	0.43 [0.05;3.79]	45 (50.56)	36 (63.16)	+ 24.91	1.68 [0.85;3.31]	59 (56.19)	42 (64.62)	+ 14.99	1.42 [0.75;2.69]
General psychiatry	–	–	–	–	13 (14.61)	8 (14.04)	− 3.91	0.95 [0.37;2.47]	13 (12.38)	8 (12.31)	− 0.59	0.99 [0.39;2.54]
Psychosomatic/Psychotherapy	< 5	< 5	–	–	32 (35.96)	12 (21.05)	− 41.45	0.47 [0.22;1.03]	33 (31.43)	13 (20.00)	− 36.36	0.55 [0.26;1.14]
Treatment duration (days)^1^, MW (± SD)	73.75 (51.36)	46.63 (42.70)	− 36.78	0.55	69.16 (74.88)	66.91 (65.89)	− 3.25	0.03	69.25 (71.53)	64.42 (63.59)	− 6.99	–
Duration until first outpatient contact after discharge (days)^1^, MW (± SD)												
General practitioner	90.70 (115.47)	75.29 (81.51)	−16.99	0.16	76.11 (124.95)	94.80 (144.83)	+ 24.57	0.14	77.85 (123.29)	91.96 (136.92)	+ 18.13	0.11
Pediatrician	36.17 (32.99)	–	–	–	55.16 (84.04)	29.53 (37.71)	− 46.47	0.39	51.53 (75.50)	26.09 (36.03)	− 49.37	–
Child and adolescent psychiatrist	15.80 (11.17)	65.33 (85.92)	+ 313.50	1.06	62.08 (97.77)	47.68 (72.93)	− 23.20	− 0.17	56.44 (92.80)	50.79 (74.28)	− 10.00	0.07
Child and adolescent psychotherapist	76.89 (68.13)	114.00 (175.64)	+ 48.27	0.40	76.85 (134.60)	40.08 (41.25)	− 47.85	− 0.37	76.86 (125.80)	53.94 (84.90)	− 29.82	0.21

*CI* confidence interval, *n* number of cases; Cohen’s |d|: small effect: ≥ 0.2. medium effect: ≥ 0.5. large effect: ≥ 0.8; F40, Phobic anxiety disorders; F41, Other anxiety disorders; F93, Emotional disorders with onset specific to childhood. Odds Ratios (OR) calculated as effect estimates based on the odds of the respective time period, change in the probability of occurrence by OR from pre- to intra-COVID; Δ%, %-change

^1^ The values refer to the population of patients with at least one hospitalization

^2^ The values refer to the population of patients with at least one hospitalization and at least one outpatient contact with one of the professional groups after their first discharge

## Discussion

We observed reductions in BN prevalence and incidence among the base population of children and adolescents in Germany during COVID-19. These reductions may be attributed to the overburdened care situation and reduced service utilization. Particularly, patients from low socioeconomic backgrounds may have been adversely affected by inadequate diagnostic and care opportunities [4]. However, this process would indicate an increase in unregistered cases. The observed rise in the quarterly BN incidence during the COVID-19 pandemic among adolescents supports this hypothesis partially. The increase in the quarterly incidence within the base population is particularly evident during the most restrictive and prolonged period of lockdown in Germany (November 2020 to March 2021). During that time, BN symptoms may have been more visible at home, leading to earlier interventions and a subsequent rise in the service utilization and administrative recording of BN incidence. Accordingly, a systematic review reported on time-specific deteriorations in ED symptomatology being more pronounced during lockdowns [3] and therefore, suggesting that such deteriorations may have become more difficult to conceal. Deteriorations during lockdown were attributed to restricted access to care, social isolation, or loss of control and structure [3]. These findings can also explain the rise in the quarterly incidence within the base population during a subsequent phase when restrictions were eased, but only for those who had been recovered, vaccinated or tested negative for SARS-CoV-2 (from August 2021) [15]. This rise in the quarterly BN incidence within the base population may either be attributed to enhanced safety using and access to health care services or to the recurrence of psychosocial stressors, which are a significant contributing factor in BN.

Nevertheless, it is unclear if there was also a quarterly rise in the BN prevalence within the base population. Reports from Germany on the deterioration of BN symptoms such as increases in the frequency of binge-eating and self-induced vomiting [16], suggest an increase in the BN prevalence since the start of the pandemic. Regarding these findings, the observed decline in prevalence throughout the whole COVID-19 period in our study appears to be contradictory. However, former inpatients also reported a decrease of psychotherapy use [16], possibly resulting in underreporting of BN prevalence. Numerically, we found a reduction in outpatient visits to general practitioners, an increase of BN in inpatient departments of child and adolescent psychiatry, and a shorter inpatient treatment duration, presumably indicating a reallocation of care distribution resources, which could also result in an underreporting of BN prevalence within the base population.

Alternatively, the pronounced visibility of BN symptoms and restrictions in health service utilization may have enhanced family interventions for existing BN diagnoses prior to COVID-19, which subsequently resulted in an actual decline in registered cases during the pandemic and therefore, the observed decline in the BN prevalence in the base population. Moreover, reduced psychosocial stressors due to infection prevention measures might have been beneficial for existing BN symptoms. Nevertheless, virtual social contacts were also documented as helpful coping mechanisms [16]. Further research findings indicating a decline in ED-specific symptoms but not in depressive or anxiety symptoms in BN [17] suggest a potential diagnostic crossover. Furthermore, patients with AN in Germany reported an increase in ED-related cognitions, such as fear of gaining weight, and shape or eating concerns [18]. As possible reasons, these patients suggested boredom, less distraction from these thoughts or an increase in social media use, among others [18]. As these ED-related cognitions are also present in BN and not need to be recorded for the diagnosis, children and adolescents with BN might have switched from BN-specific (e.g. self-induced vomiting, binge eating) to AN-specific (e.g. restrictive eating, hyperactivity) coping mechanisms as a COVID-19-related adaptation with AN-specific disease manifestations being easier to conceal. A diagnostic cross-over from BN to AN might be a reasonable explanation for the decreases in the BN-prevalence within the base population, while other studies reported increases in the AN prevalence during COVID-19 [7, 19]. However, Spettigue et al. [20] also found a rise in purging behavior among individuals with AN, which is rather a defining characteristic of BN and usually less pronounced in AN. Furthermore, given the potentially fatal consequences of AN, the reported increases in prevalence may indicate a need for more extensive service resources. Accordingly, a recent meta-analysis found COVID-related deteriorations of ED symptoms specifically in AN [6]. However, another meta-analysis of studies published between 1980 and 2021 on the outcomes of ED treatment revealed that approximately 2% of BN patients exhibited a diagnostic shift to AN [21], indicating a clinically relevant prevalence shift. The diagnostic cross-over in untreated or unregistered cases may be even higher. There may also have been a shift from BN to BED, also as a result of adaptations made due to the heightened visibility or more difficult concealment of the compensatory behaviors for binge-eating (i.e., purging) that are typically associated with BN but rarely present in BED.

In summary, the rise in quarterly incidence in adolescents within the base population since the COVID-19 pandemic onset suggests a deterioration/chronification in the long-term, or delayed diagnosis and thus a potential backlash that researchers and health care providers need to consider.

## Strength and limits

To our knowledge, this study represents the first examination of BN among children and adolescents in Germany during the COVID-19 pandemic using claims data from statutory health insurances. This approach allows for the analysis of real-world epidemiological trends and healthcare utilization in a large population on which data remains limited. The findings are not influenced by non-responses or recall bias, as the data were collected prospectively without relying on patients’ memory or voluntary participation.

However, it is important to consider the following limitations when interpreting the study results. Analyses are based on claims data and restricted to diagnoses; verifying the validity of coded diagnoses is not possible. The strict case definition may have underestimated BN prevalence and incidence within the base population. Data on frequency and severity of symptoms of BN and of comorbidities are not part of the analyzed database. The privately insured population is likely to be of a higher socioeconomic status and lower morbidity rate [22]. As our analyses were limited to data from statutorily insured children and adolescents, the findings cannot be generalized to the entire group of children and adolescents aged 10.0–17.9 years in Germany. Moreover, given the relatively small sample sizes, it was not possible to further differentiate by SES subgroups, as this three-level stratification would have resulted in sample sizes that would be too small, with some missing data due to critically small cell sizes, preventing a meaningful interpretation of the results. Due to the exploratory nature of the analyses, we did not correct for multiple testing. Some analyses suffered from small sample sizes. Finally, we did not stratify the analyses by sex (female vs. male) due to the small sample sizes in general and specifically in the male population. It is well-documented that females are more affected by BN than males [23, 24], but males also showed divergent trends to females with declining incidence during COVID-19 [4]. To enhance explanatory and statistical power, we therefore decided to combine both sexes into one sample.

## Conclusion

The observed decline in diagnosed and treated BN cases and the positive trend in quarterly incidence within the base population may be attributed to an increase in unregistered cases resulting from the overburdened care situation that emerged with the onset of the COVID-19 pandemic. The time-specific increases in quarterly incidence within the base population during lockdown and a subsequent phase of easing of preventive measures may serve to highlight the role of social isolation, loss of control and structure, and/or psychosocial stressors in BN, while also underscoring the potential impact of the COVID-19-related restrictions on social and public life on children’ and adolescents’ mental health. Additionally, the observed decline in BN prevalence within the base population is inconsistent with prior findings on deteriorations in the frequency of binge-eating and self-induced vomiting [16]. However, our data on the utilization of health care services, as well as previous results from Germany [16], suggest reduced utilization regardless of the presence or severity of BN symptoms. It is therefore imperative that researchers and healthcare providers remain aware of the potential for a backlash and deterioration/chronification of BN symptoms in children and adolescents.

## What is already known on this subject?

Previous studies reported an increase in the incidence of EDs and deteriorations in ED symptoms in AN and BN [3, 4]. Hospitalizations due to (atypical) AN increased as well [7], while international results on BN indicate a decline in the proportion of BN among ED-related hospitalizations [8]. As studies on BN are scarce and reveal divergent findings compared to EDs and AN, this study aimed to contribute to existing research about specific EDs during COVID-19 by also including data of the epidemiology, comorbidity and care of children and adolescents with BN in Germany drawn from a representative database.

## What this study adds?

This study provides insight into the administrative BN prevalence and incidence in children and adolescents in Germany pre-vs. intra-COVID-19 pandemic as well as utilization of health care services by those affected. The findings can inform practitioners of the potential for a backlash and deterioration and/or chronification of BN symptoms in children and adolescents, and policy makers about epidemiological and utilization patterns during/after phases of restrictions on social and public life and their consequences on mental health in children and adolescents.

## Supplementary Information

Supplementary file 1

## Data Availability

The analyzed dataset is available from the corresponding author on reasonable request.

## Abbreviations

ADHD: Attentional-deficit/hyperactivity disorder
BED: Binge-eating disorder
BN: Bulimia nervosa
EBM: Standardized evaluation scale [Einheitlicher Bewertungsmaßstab]
ED: Eating disorder
ICD: International Classification of Diseases
OCD: Obsessive–compulsive disorder
