# Supplementary material for: Decreasing prevalence or increase in unregistered cases of bulimia nervosa in children and adolescents in Germany? A comparison using representative claims data pre- vs. intra-COVID-19 pandemic
Source: Eat Weight Disord. 2025 Mar 21;30(1):30. doi: 10.1007/s40519-025-01738-z (PMC11928349; doi:10.1007/s40519-025-01738-z)
Supplement: Supplementary file 1 [file 40519_2025_1738_MOESM1_ESM.docx]

**Decreasing Prevalence or Increase in Unregistered Cases of Bulimia Nervosa in Children and Adolescents in Germany? A Comparison Using Representative Claims Data Pre- vs. Intra-COVID-19 Pandemic**

Eating and Weight Disorders – Studies on Anorexia, Bulimia and Obesity

Leickert J, Zillmer S, Bachmann C, Vivirito A, Enders D, Correll CU, Jaite C

Corresponding author: Charlotte Jaite, Department of Clinical Psychology and Psychotherapy in Childhood and Adolescence, University of Hildesheim, Hildesheim, Germany, jaite@uni-hildesheim.de

**Electronic supplementary material 1**

Psychiatric comorbidities in bulimia nervosa in girls and boys pre- vs. intra-COVID, stratified by age groups.

| **Age groups** | **10-13 years** | | | | **14-17 years** | | | | **10-17 years** | | | |
| --- | --- | --- | --- | --- | --- | --- | --- | --- | --- | --- | --- | --- |
|  | **Pre-**  **COVID** | **Intra-COVID** | **Δ%** | **OR**  **[95%CI]** | **Pre-**  **COVID** | **Intra-COVID** | **Δ%** | **OR**  **[95%CI]** | **Pre-**  **COVID** | **Intra-COVID** | **Δ%** | **OR**  **[95%CI]** |
| **At least one psychiatric comorbidity**, n (%) | 33 (86.84) | 29 (90.63) | +4.36 | 1.46 [0.32;6.67] | 186 (80.87) | 148 (83.62) | +3.40 | 1.21 [0.72;2.02] | 219 (81.72) | 177 (84.69) | +3.64 | 1.24 [0.76;2.02] |
| **Organic mental disorders (F00-09)***,* n (%) | - | - | - | - | <5 | - | - | - | <5 | - | - | - |
| **Substance use disorders (F10-F19),** n (%) | <5 | <5 | - | - | 24 (10.43) | 15 (8.47) | -18.79 | 0.79  [0,40; 1,56] | 26 (9.70) | 18 (8.61) | -11.23 | 0.88 [0.47;1.65] |
| Alcohol abuse (F10) | <5 | - | - | - | 9  (3.91) | 9  (5.08) | +29.94 | 1.32 [0.51;3.39] | - | - | - | - |
| Cannabis use (F12) | <5 | <5 | - | - | 7  (3.04) | <5 | - | - | - | <5 | - | - |
| **Schizophrenia spectrum disorder (F20-F29)** | <5 | <5 | - | - | 5  (2.17) | 5  (2.82) | +29.94 | 1.31 [0.37;4.59] | 6  (2.24) | 6  (2.87) | +28.23 | 1.29 [0.41;4.06] |
| Schizophrenia (F20) | - | - | - | - | - | - |  | - | - | - | - | - |
| Acute psychosis (F23) | - | - | - | - | <5 | <5 | - | - | <5 | <5 | - | - |
| **Mood disorders (F30-F39)** | 19 (50.00) | 14 (43.75) | -12.50 | 0.78 [0.30;2.00] | 143 (62.17) | 113 (63.84) | +2.68 | 1.07 [0.72;1.61] | 162 (60.45) | 127 (60.77) | +0.53 | 1.01 [0.70;1.47] |
| Bipolar disorder (F30-F31) | - | - | - | - | <5 | <5 | - | - | <5 | <5 | - | - |
| Depressive disorder (F32-F33, F34.1) | 19 (50.00) | 14 (43.75) | -12.50 | 0.78 [0.30;2,00] | 142 (61.74) | 113 (63.84) | +3.41 | 1.09 [0.73;1.64] | 161 (60.07) | 127 (60.77) | +1.15 | 1.03 [0.71;1.49] |
| **Neurotic, stress and somatoform disorders (F40-F48, F93)** | 23 (60.53) | 25 (78.13) | +29.08 | 2.33 [0.81;6.73] | 137 (59.57) | 107 (60.45) | +1.49 | 1.04 [0.70;1.55] | 160 (59.70) | 132 (63.16) | +5.79 | 1.16  [0.80;1.68] |
| Anxiety and emotional disorders (F40, F41, F93) | 9 (23.68) | 19 (59.38) | +150.69 | **4.71****  **[1.68;13.17]** | 59 (25.65) | 64 (36.16) | +40.96 | **1.64***  **[1.07;2.51]** | 68 (25.37) | 83 (39.71) | +56.52 | **1.94*****  **[1.31;2.86]** |
| Obsessive-compulsive disorder (F42) | - | <5 | - | - | 8  (3.48) | 8  (4.52) | +29.94 | 1.31 [0.48;3.57] | 8  (2.99) | 11 (5.26) | +76.32 | 1.81 [0.71;4.57] |
| Post-traumatic stress disorder (F43.1, F43.8, F43.9) | <5 | 5 (15.63) | - | - | 30 (13.04) | 20 (11.30) | -13.37 | 0.85  [0.46;1.55] | 33 (12.31) | 25 (11.96) | -2.86 | 0.97  [0.56;1.68] |
| Adjustment disorder (F43.0, F43.2) | 14 (36.84) | 9 (28.13) | -23.66 | 0.67 [0.24;1.85] | 55 (23.91) | 43 (24.29) | +1.59 | 1.02  [0.65;1.61] | 69 (25.75) | 52 (24.88) | -3.36 | 0.96  [0.63;1.45] |
| Dissociative disorders (F44) | <5 | <5 | - | - | 8  (3.48) | <5 | - | - | 10 (3.73) | - | -100.00 | - |
| Somatoform disorders (F45) | 10 (26.32) | 7 (21.88) | -16.88 | 0.78 [0.26;2.37] | 55 (23.91) | 22 (12.43) | -48.02 | **0.45** [0.26;0.77]** | 65 (24.25) | 29 (13.88) | -42.79 | **0.50** [0.31;0.81]** |
| **Personality disorders (F60-F69)** | 7 (18.42) | 7 (21.88) | +18.75 | 1.24 [0.38;4.01] | 52 (22.61) | 31 (17.51) | -22.53 | 0.73 [0.44;1.19] | 59 (22.01) | 38 (18.18) | -17.41 | 0.79 [0.50;1.24] |
| **Intellectual disability (F7)** | - | - | - | - | <5 | - | - | - | <5 | - | - | - |
| **Other developmental disorders (F80-F83)** | 8 (21.05) | 6 (18.75) | -10.94 | 0.87 [0.27;2.82] | 13 (5.65) | 12 (6.78) | +19.95 | 1.21 [0.54;2.73] | 21 (7.84) | 18 (8.61) | +9.91 | 1.11 [0.57;2.14] |
| **Autism spectrum disorders (F84.x exkl. F84.2-F84.4)** | <5 | - | - | - | <5 | <5 | - | - | - | <5 | - | - |
| Childhood autism (F84.0) | <5 | - | - | - | <5 | <5 | - | - | <5 | <5 | - | - |
| Asperger syndrome (F84.5) | <5 | - | - | - | <5 | <5 | - | - | <5 | <5 | - | - |
| **Attention-deficit/hyperactivity disorder (F90.0 + F98.8)** | <5 | 7 (21.88) | - | - | 18 (7.83) | 20 (11.30) | +44.38 | 1.50 [0.77;2.93] | 22 (8.21) | 27 (12.92) | +57.37 | 1.66 [0.92;3.01] |
| Combined type (F90.0) | <5 | 6 (18.75) | - | - | 11 (4.78) | 14 (7.91) | +65.38 | 1.71 [0.76;3.86] | 13 (4.85) | 20 (9.57) | +97.28 | **2.08***  **[1.01;4.28]** |
| Inattentive type (F98.8) | <5 | <5 | - | - | 11 (4.78) | 8  (4.52) | -5.50 | 0.94 [0.37;2.39] | - | - | - | - |
| **Oppositional defiant disorder/conduct disorder (F91, F90.1, F92)** | 8 (21.05) | 5 (15.63) | -25.78 | 0.69 [0.20;2.38] | 17 (7.39) | 12 (6.78) | -8.28 | 0.91 [0.42;1.96] | 25 (9.33) | 17 (8.13) | -12.80 | 0.86  [0.45;1.64] |
| **Attachment disorders (F94.x exkl. F94.0)** | - | <5 | - | - | <5 | <5 | - | - | <5 | <5 | - | - |
| **Tic disorders (F95)** | <5 | - | - | - | - | <5 | - | - | <5 | <5 | - | - |

*Note.* CI, confidence interval; n, number of cases; Odds Ratios (OR) calculated as effect estimates based on the odds of the respective time period, change in the probability of occurrence by OR from pre- to intra-COVID; Δ%, %-change; * p < .05; ** p < .01; *** p < .001.
